# Key role of microsurgical dissections on cadaveric specimens in neurosurgical training: Setting up a new research anatomical laboratory and defining neuroanatomical milestones

**DOI:** 10.3389/fsurg.2023.1145881

**Published:** 2023-03-09

**Authors:** Arianna Fava, Nicola Gorgoglione, Michelangelo De Angelis, Vincenzo Esposito, Paolo di Russo

**Affiliations:** ^1^ Department of Neurosurgery, IRCCS Neuromed, Pozzilli, Italy; ^2^ Department of Neuroscience, Sapienza University, Rome, Italy

**Keywords:** neurosurgical training, anatomical dissection, anatomy laboratory, cadaveric specimen, skull base technique, microanastomosis

## Abstract

**Introduction:**

Neurosurgery is one of the most complex surgical disciplines where psychomotor skills and deep anatomical and neurological knowledge find their maximum expression. A long period of preparation is necessary to acquire a solid theoretical background and technical skills, improve manual dexterity and visuospatial ability, and try and refine surgical techniques. Moreover, both studying and surgical practice are necessary to deeply understand neuroanatomy, the relationships between structures, and the three-dimensional (3D) orientation that is the core of neurosurgeons' preparation. For all these reasons, a microsurgical neuroanatomy laboratory with human cadaveric specimens results in a unique and irreplaceable training tool that allows the reproduction of patients' positions, 3D anatomy, tissues' consistencies, and step-by-step surgical procedures almost identical to the real ones.

**Methods:**

We describe our experience in setting up a new microsurgical neuroanatomy lab (IRCCS Neuromed, Pozzilli, Italy), focusing on the development of training activity programs and microsurgical milestones useful to train the next generation of surgeons. All the required materials and instruments were listed.

**Results:**

Six competency levels were designed according to the year of residency, with training exercises and procedures defined for each competency level: (1) soft tissue dissections, bone drilling, and microsurgical suturing; (2) basic craniotomies and neurovascular anatomy; (3) white matter dissection; (4) skull base transcranial approaches; (5) endoscopic approaches; and (6) microanastomosis. A checklist with the milestones was provided.

**Discussion:**

Microsurgical dissection of human cadaveric specimens is the optimal way to learn and train on neuroanatomy and neurosurgical procedures before performing them safely in the operating room. We provided a “neurosurgery booklet” with progressive milestones for neurosurgical residents. This step-by-step program may improve the quality of training and guarantee equal skill acquisition across countries. We believe that more efforts should be made to create new microsurgical laboratories, popularize the importance of body donation, and establish a network between universities and laboratories to introduce a compulsory operative training program.

## Introduction

Neurosurgery is one of the most complex and highly demanding surgical disciplines. Intensive and long training is required to acquire a solid theoretical background, deep anatomical knowledge, hand–eye coordination, manual dexterity, and complex technical skills, in addition to controlling physiologic and psychological tremors. Young neurosurgeons must be aware of the intricate three-dimensional neuroanatomy (3D), the psychomotor abilities, the fatigue related to long operative surgeries, and accurate and safe manipulation in deep and narrow operative corridors, which can affect the surgical performance and results (1–3). For all these reasons, neurosurgical laboratories with human cadaveric specimens result in a unique and irreplaceable training tool for developing and refining anatomical knowledge, dexterity, technical skills, and surgical procedures before performing them on a living patient (4–6). The use of a real human specimen gives the possibility to reproduce the actual procedure, starting from the head positioning to the step-by-step surgery facing narrow corridors, fragile neurovascular structures, and a delicate brain surface, which can give an idea of how gentle and accurate the dissection has to be during the live surgery (4, 6, 7). Nevertheless, permanent neurosurgical laboratories are not widespread all over the world due to strict requirements and permissions, ethical and legal issues, and the high costs related to equipment, materials, and specimens. On the other hand, in the last few decades, medicolegal issues in the medical field and, in particular, neurosurgical practice have increased (8, 9). For these reasons, neurosurgical training has become crucial, with increasing interest in it, to train the next generation of neurosurgeons before practicing on living patients to reduce perioperative complications (10, 11). In this scenario, in recent years, the European Union has increased its financial support to settle up new anatomical and surgical laboratories, and, in parallel, the Italian government has unlocked some restrictions on body donation (12, 13). Convinced of the irreplaceable value of a cadaver lab, in this article we describe our experience in setting up a new microsurgical neuroanatomy lab (IRCCS Neuromed, Pozzilli, Italy), focusing on the development of training activity programs and microsurgical milestones useful to train the next generation of surgeons.

## Materials and methods

We set up a microsurgical neuroanatomy laboratory, “Laboratorio di neuroanatomia G. Cantore” (Centro di Medicina Necroscopica-Unità di Chirurgia Formativa), at Parco Tecnologico IRCCS Neuromed in Pozzilli (Italy), according to the Italian requirements (Law no. 10, 10 February 2020; G.U., 4 March 2020). The laboratory and all its activities on human and animal specimens were approved by the ethical committee of IRCCS Neuromed. Embalmed and latex-injected human cadaveric heads have been used for dissections, while the human placenta has been furnished by the Obstetrics and Gynecology Department of the Istituto Clinico Mediterraneo (ICM, Salerno, Italy) as a scientific donation after birth, and Sprague–Dawley rats were provided by the Neuromed stabularium according to the Italian law on laboratory animal welfare (D.lgs. 26/2014). The human placenta was prepared as reported elsewhere (14). A dedicated veterinarian took care of the rodents, proceeding with anesthesia, analgesia, and immobilization during the courses. All the required materials and instruments are listed in Table 1. Moreover, a permanent neuronavigation system (Treon, Medtronic) is present in the lab for measurements and anatomical verification during research activities.

**Table 1:** Required materials and instruments.

	Transcranial approaches	Endoscopic approaches	Vascular anastomosis	White matter dissection
Tools	Microscope	Endoscope	Microscope	—
	Exoscope		Exoscope	—
Specimens	Human-injected cadaveric heads	Human-injected cadaveric heads	Silicone tubes	Human brain hemispheres
			Chicken wings	
			Human placenta	
			Human cadaveric head	
			Rats	
Materials	—	—	SP + dyes	—
	Drapes	Drapes	Drapes	Drapes
	Gauzes	Gauzes	Cotton fioc	Gauzes
	Syringes	Syringes	Syringes	—
	Stitches	—	Stitches	
	Mayfield	Mayfield	Approximators	—
	—	—	Pins	—
Instrumentation	Suction	Suction	—	—
	Engine, drills, perforator	Engine, E. drills	—	—
	Scalpel	E. scalpel	Microscalpel	Tongue depressor
	Retractors	—	Retractors (rats)	—
	Macrosets^a^	Endoscopic set	Macroset^a^	Dissector
	Microsets^b^	—	Bypass set^c^	—

SP, physiological solution; E, endoscopic.

^a^ Two pairs of forceps, scissors, rongeurs, dissectors, backhaus clamps, klemmer forceps.

^b^ (Bayonet-shaped) microscissors, two pairs of microforceps.

^c^ Needle holders, tying forceps, jeweler forceps.

The training program for neurosurgical residents has been developed considering the progressive competency acquired in each year of residency, from basic techniques and procedures to complex anatomy and approaches. Four types of dissection courses have already been organized at our laboratory for neurosurgical residents and young neurosurgeons: (a) basic techniques and approaches using the microscope; (b) the 3D exoscope; (c) transcranial and endoscopic skull base approaches; and (d) microvascular anastomosis on the human placenta and rats. Furthermore, an ongoing anatomical research activity is currently being performed.

A review of the literature on anatomy and dissection guides has been done to provide a list of “suggested references” for the preparation of trainees.

## Results

**Figure 1:**
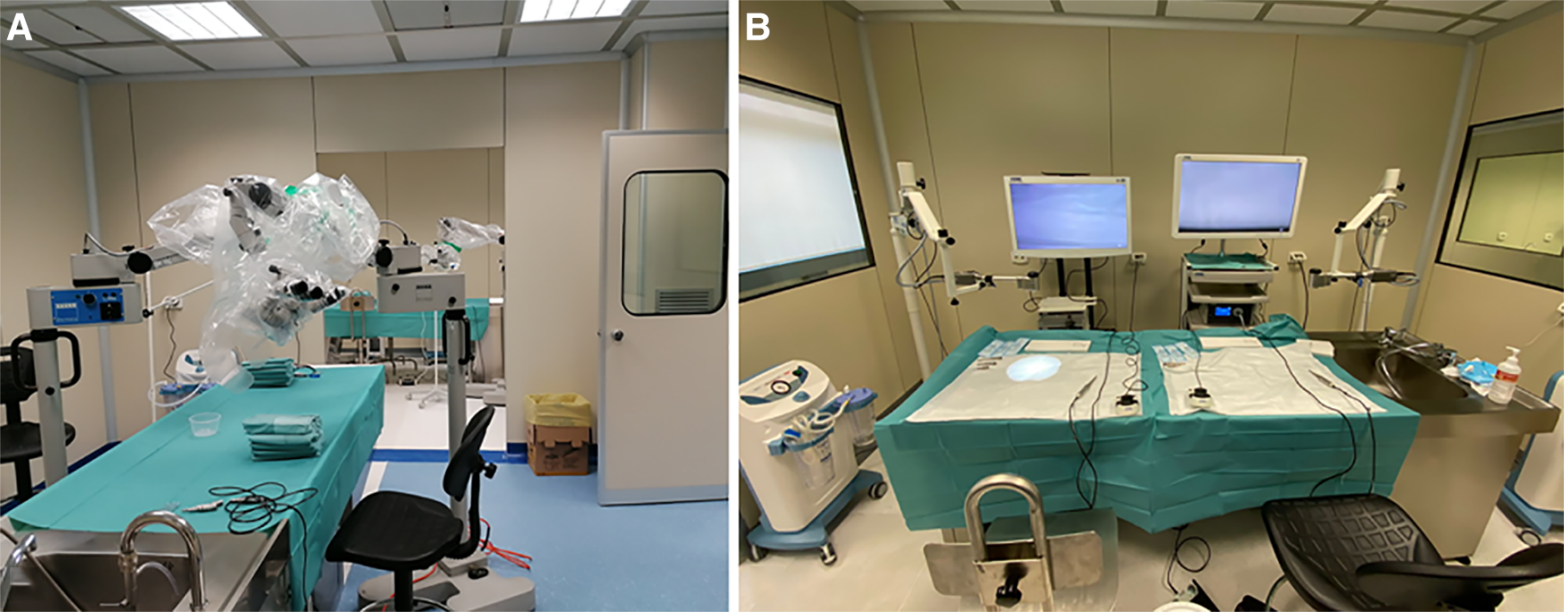
Workstations at “Laboratorio di neuroanatomia G. Cantore.”

Six competency levels were designed according to the year of residency (PGY, from 1 to 5): (1) soft tissue dissections, bone drilling, and microsurgical suturing; (2) basic craniotomies and neurovascular anatomy; (3) white matter dissection; (4) skull base transcranial approaches; (5) endoscopic approaches; (6) vessel preparation on placenta and rats; and (7) microanastomosis. For each level, a checklist with milestones is provided in Table 2. Figure 1 shows the organization of our lab.

**Table 2:** Proposed milestone checklist for training.

	Level 1 (neurosurgical basics)	Level 2 (neurosurgical basics)	Level 3 (white matter)	Level 4 (skull base)	Level 5 (endoscopic skull base)	Level 6 (vascular anastomosis)
Tools	Macro microscope exoscope	Macro microscope exoscope	Macro microscope exoscope	Microscope exoscope	Endoscope	Microscope exoscope
Soft tissues	– Dissection of the facial nerves, SON, GON, GAN, STA, OA – Interfascial dissection of the TM – Dissection of the nuchal and neck muscles			– Interfascial dissection of the TM – Dissection of the nuchal and neck muscles	– Nasoseptal flap	
Bone	– Common Burr holes	– Pterional, subtemporal, parasagittal, retrosigmoid, suboccipital, craniotomies – Sphenoid wing, temporal base, TS-SS, SSS drilling		– FTOZ, anterior/posterior/combined petrosal, far lateral, transcondylar, anterolateral approaches	– Sphenoidectomy – Ethmoidectomy – Medial approaches: planum, sella, clivus, CCJ – Lateral approaches: maxillectomy, transpterygoid, orbital, middle fossa, petrous apex surgery	
Dura mater		– Dura mater opening – Macro- and microscopic suturing		– Interdural dissection from the MOB cut (peeling of the LWCS), from V3 (Kawase triangle) – Tentorial cutting	– Sellar, suprasellar, CS, clival, temporal dura mater opening – MOB cut – periorbit opening	
Neuroanatomy	– Superficial neurovascular anatomy	– Sylvian and interhemispheric fissures splitting – ICA and branch dissection – Neurovascular triangles – Sinuses – CPA anatomy	– Sulci and gyri, brainstem, cerebellum anatomy – U fibers, operculi, insula, CR, SLF, sagittal stratum, EC, UF, IFOF, IC, putamen, GP, APS, ventricular ependyma, CN, TH, optic radiations, cingulum, CC, fornix, hypothalamus	– Cranial nerves from the brainstem to foramina – CS, middle fossa triangles – Supra- and infratentorial anatomy – Nasal anatomy – Sphenoid sinus anatomy – Pituitary gland, optic chiasm, pituitary stalk, third ventricle, frontal lobes, CS, orbit, ICA and branches, brainstem, VA, BA, branches, cranial nerves		
Vessels	– Dissection of all the STA	– ICA, VA, BA, and branches		– ICA, VA, BA, and branches	– ICA, VA, BA, and branches	Vessels dissection
Sutures	– Macro- and microscopic sutures on soft tissues	– Macro- and microscopic sutures on the dura mater			– Mucosa suture	Microanastomosis EEA ESA SSA

SON, supraorbital nerve; GON, greater occipital nerve; GAN, greater auricular nerve; STA, superficial temporal artery; OA, occipital artery; TM, temporalis muscle; TS, transverse sinus; SS, sigmoid sinus; SSS superior sagittal sinus; ICA, internal carotid artery; CPA, cerebellopontine angle; CR, corona radiata; EC, extreme capsule; UF, uncinate fasciculus; IC, internal capsule; GP, globus pallidus; APS, anterior perforated substance; CN, caudate nucleus; TH, thalamus; CC, corpus callosum; FTOZ, frontotemporal orbito-zygomatic; MOB, meningo-orbital band; LWCS, lateral wall of the cavernous sinus; CS, cavernous sinus; VA, vertebral artery; BA, basilar artery; CCJ, craniocervical junction; EEA, end-to-end anastomosis; SSA, side-to-side anastomosis; ESA, end-to-side anastomosis.

### Level 1: soft tissue dissections, bone drilling, and microsurgical suturing (PGY 1–2)

Level 1 represents the surgical basics for PGY1. The junior resident should know the different myocutaneous layers, their relationship with nerves and vessels, craniometric points, and differences between cortical and cancellous bones. At this level, the resident must become familiar with the Mayfield head holder, macroscopic instruments, dissection techniques, engine functioning, bone drilling, and macro and microscopic suture techniques.

### Level 2: basic craniotomies and neurovascular anatomy (PGY 2–3)

Level 2 corresponds to PGY2 and PGY3. The resident should be able to perform basic craniotomies, that is, pterional, subtemporal, parasagittal, retrosigmoid, and suboccipital approaches. They must perform Sylvian and interhemispheric fissure openings and know the target anatomy exposed during each approach. The resident must become familiar with bony drilling without injuring neurovascular structures.

### Level 3: white matter dissection (PGY 3–4)

Level 3 represents the anatomical knowledge of white matter for PGY3 and PGY4. Knowledge of the anatomy of the cerebral surface, white matter fibers, deep nuclei, brainstem, and cerebellum is mandatory. At this level, the resident becomes confident enough in white matter dissection to dissect the main structures.

### Level 4: skull base transcranial approaches (PGY 4–5)

Level 4 represents the advanced transcranial craniotomies for skull-base pathologies. This level is addressed to PGY4 and PGY5. The resident should be able to perform those approaches and recognize the extra- and intradural anatomies. Gentle interdural dissection, bony drilling in deep and narrow fields, and cutting the tent without injuring neurovascular structures are important technical skills.

### Level 5: endoscopic transnasal approaches (PGY 4–5)

Level 5 is addressed to PGY4 and PGY5 and corresponds to endoscopic transnasal approaches. The resident should learn the anatomy of the nasal cavity and its relationship with the maxillary, orbital, ethmoidal, sphenoidal, clival, and craniocervical junction (CCJ) compartments. Preparation of the nasoseptal flap and the transsphenoidal approach to the sella is the first step, followed by extended medial and lateral approaches. The resident must become familiar with the endoscope and one-hand dissection. Finally, an endoscopic mucosal suture should be attempted.

**Figure 2:**
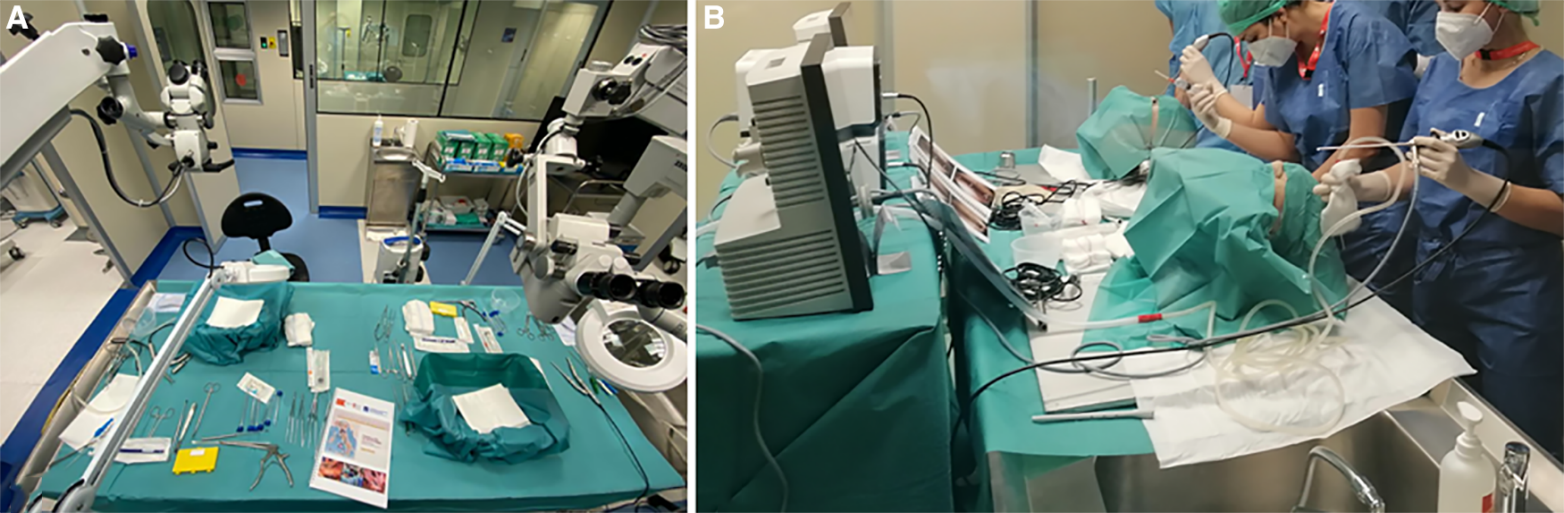
Arrangement of the stations during our (**A**) microsurgical and (**B**) endoscopic courses.

Figure 2 shows the arrangement of the stations during our microsurgical and endoscopic courses.

### Level 6: vessel preparation and microanastomosis on inert materials, chicken wings, human placenta, human cadavers, and rats (PGY 5)

**Figure 3:**
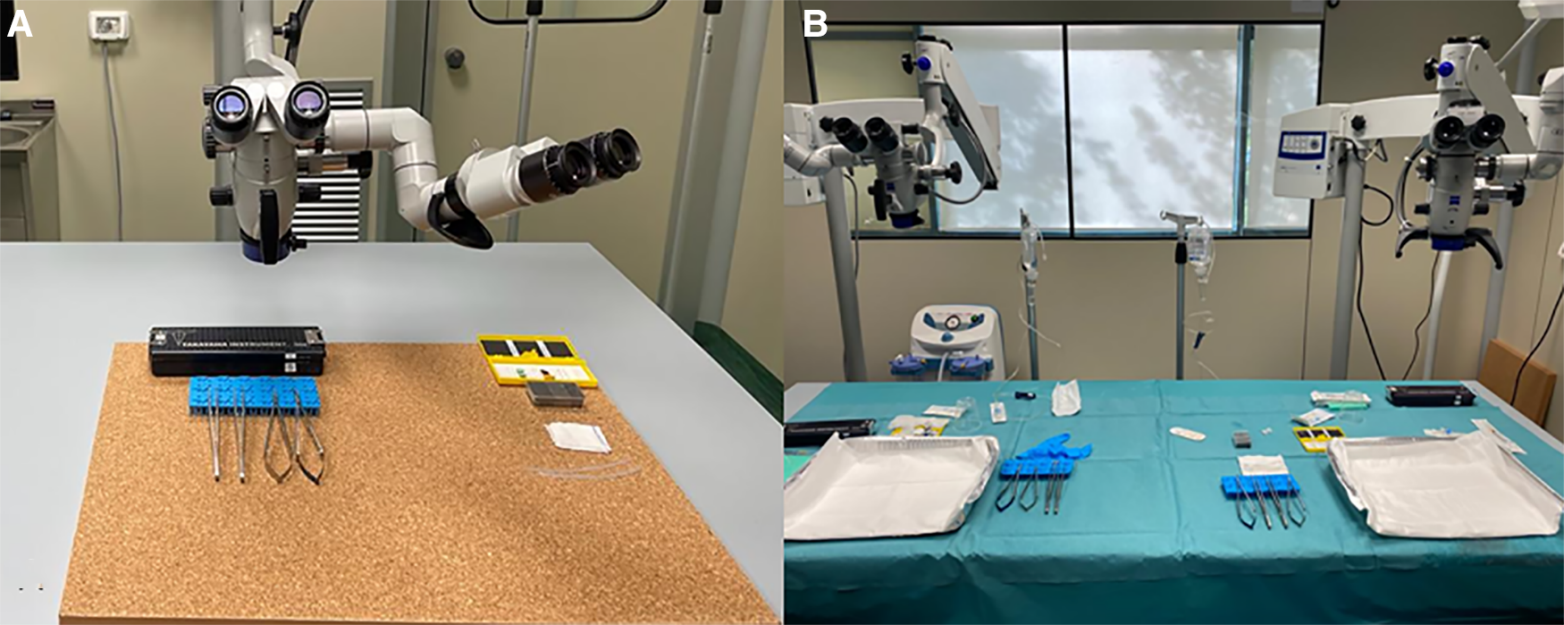
Workstation for microanastomosis on (**A**) inert materials and (**B**) wet specimens.

**Figure 4:**
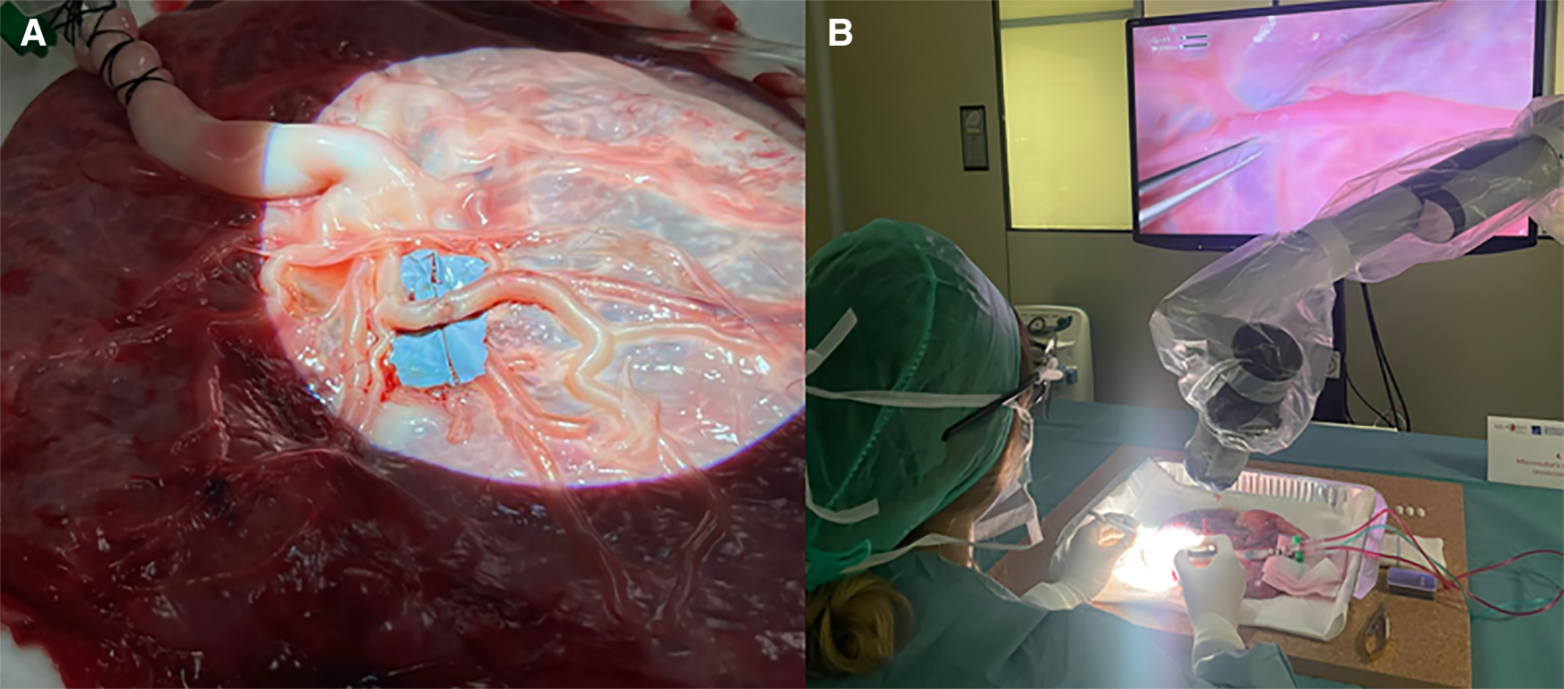
(**A**) Preparation of human placenta for microanastomosis with cannulation of the umbilical arteries and vein. After washing arteries and veins with clean water to remove blood clots, colored saline solution is used to continuously fill the vessels mimicking the blood. (**B**) Vascular dissection and microanastomosis on human placenta using a 3D exoscope.

**Figure 5:**
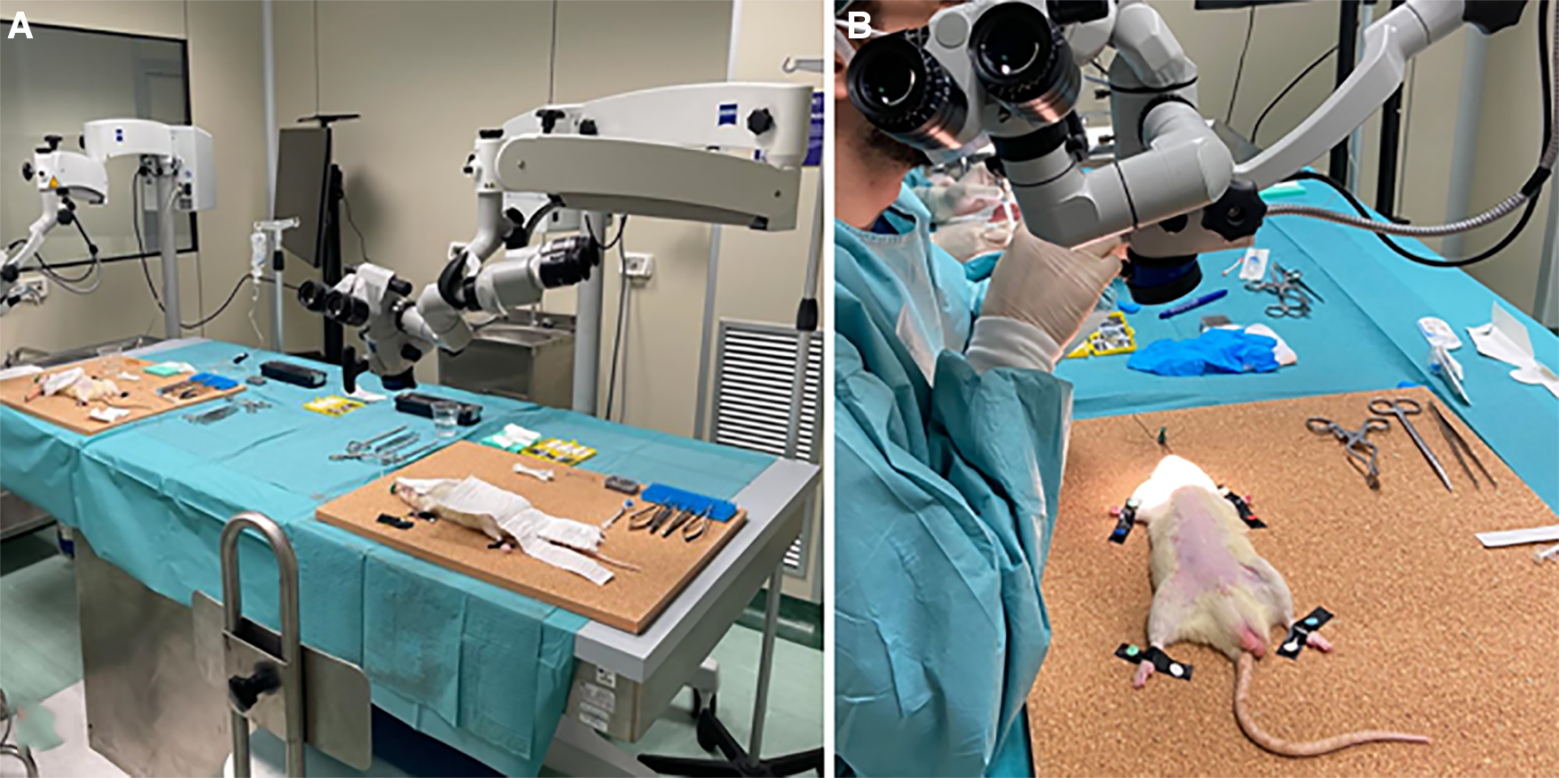
(**A,B**) Workstation to perform dissection and microanastomosis on *in vivo* rats.

Level 6 is addressed to PGY 5 and represents one of the most difficult skills to acquire in neurosurgery, namely vascular anastomosis. Starting from inert materials such as gauze and silicon tubes, the resident should train in simple knots and end-to-end, end-to-side, and side-to-side microanastomoses. Figure 3 shows the organization of the stations. Chicken wings and human placenta are used to mimic the consistency of the vessels and to train with vessel dissection. As described elsewhere (14), the human placenta is prepared and injected continuously with red and blue saline solution by cannulating the umbilical arteries and veins (Figure 4). The following step is using human cadaveric heads and *in vivo* rats. In particular, the human specimen allows for the replication of a real surgical procedure for bypass, dissecting vessels, and performing microanastomoses in deep fields. On the other side, the *in vivo* rat is the most realistic model through which it is possible to simulate bloody dissection, blood pulsation, empathy, and anxiety (Figure 5).

Finally, in Table 3, we reported a list of some useful books and articles’ references for each level of competency to guide the residents during their studies and cadaveric dissections.

**Table 3:** Suggested references for dissections.

Neurosurgical basics	White matter	Skull base	Endoscopic skull base	Vascular anastomosis
Anatomical basics and surgical approaches (15–31), temporal muscle and pterional approach (32–37), neck anatomy (38, 39), suboccipital (40), retrosigmoid (41–46), subtemporal (31, 47)	Fundamentals (48–69)	FTOZ (70–75), clinoidectomy and cavernous sinus (30, 76–80), petrosal approaches (81–86), posterolateral approaches (39, 87, 88), anterolateral approach (89–91)	(92, 93) Reconstruction (94–96)	(4, 97–100)

FTOZ, fronto-temporal orbito-zygomatic.

## Discussion

The education and training needed to become a neurosurgeon consist of a long and demanding program that consists of acquiring a solid theoretical background and clinical and surgical experience. Residents in neurosurgery have to spend a lot of time in the operating room to become familiar with surgical anatomy and techniques and to develop practical skills (1–3). Although intraoperative surgical exposure represents the fundamental way to train surgeons, several drawbacks are encountered in every country. Differences between departments, hospitals, and nations are wide, and residents often do not have the same opportunity in their education and operation room in particular (101–107). Considering the European centers, recent surveys among neurosurgical residents report very low satisfaction rates for the theoretical and practical aspects of training in some countries (101). Moreover, novel regulations on reducing working hours for residents reduce the possibilities for practical and psychophysical training, affecting the quality of the residency and prolonging the learning curve (108, 109). These controversial aspects are also critical for the increasing medicolegal issues affecting medical practice in the last few decades (8, 9). Accordingly, the impossibility to try or repeat surgical procedures makes neurosurgical training more difficult. For all these reasons, training on human cadaveric specimens results in an essential and irreplaceable tool for residents and fully-trained neurosurgeons (10). Similarly, the evolution of simulation technology applied to surgery has assumed an important role in learning anatomy, completing procedural tasks, and improving accuracy and hand–eye coordination. In recent years, 3D inert models and virtual and augmented reality have been popularized among universities and through courses to train young residents (110, 111). Nevertheless, the use of a real human specimen gives the possibility to reproduce the real procedure starting from the head positioning to the step-by-step surgery facing narrow corridors, fragile neurovascular structures, and a delicate brain surface that can give an idea of how gentle and accurate the dissection has to be during the live surgery. Last but not least, training on human cadavers differs from inert materials or simulation tools because the trainer must also manage the emotional counterpart, empathy, and respect for the human body that can approximate real surgery.

### Laboratory setup

After the introduction in Italy of Law no. 10 of 10 February 2020 entitled “Rules regarding the disposition of one's body and postmortem tissues for study, training, and scientific research purposes,” our Neurosurgical Department and IRCCS Neuromed have worked together to set up a new microsurgical neuroanatomy laboratory called “Laboratorio di neuroanatomia G. Cantore” (Centro di Medicina Necroscopica—Unità di Chirurgia Formativa) at Parco Tecnologico Neuromed in Pozzilli (Italy). Finally, our laboratory has been accredited as a no-profit laboratory for body donation with other centers in Italy. This lab has been created based on our previous experiences with neurosurgical dissections in several laboratories around the world to offer educational opportunities and training activities for residents and neurosurgeons from Italy and abroad and establish research fellowships and permanent surgical research. After the initial funding for no-profit research, a policy of reuse has been applied in our no-profit lab. The necessary equipment has been found through the recovery of disused microscopes and instruments, a donation from retired surgeons, and second-hand instruments left in the lab after courses. Expired or nonsterile materials are also routinely brought into the operative room (OR) of our hospital.

For research fellows (two for each semester) and residents, a “neurosurgical booklet,” represented in Table 2 as a checklist, is furnished to give a progressive training guide. The concept is to take all the time to perform slow and gentle step-by-step dissection to preserve tissues and understand the 3D surgical anatomy and intricate relationships between structures. Meticulous work and a sound state of mind are essential to preparing the fellow for the accuracy necessary to be a neurosurgeon. Moreover, it is essential to acquire strong neuroanatomical knowledge through transcranial and endoscopic dissections to understand the anatomy from different perspectives directly on the specimen, which is the basis for performing their research.

In parallel, fellows can observe surgical procedures to treat skull base pathologies, gliomas, or epilepsy in our department, taking inspiration to learn more about a procedure directly on the specimen or to develop new anatomical research or approaches.

### Courses

During the first year of activity, we organize six courses for neurosurgical residents and young neurosurgeons, ranging from basic to more complex: (a) basic techniques and approaches using the microscope and (b) the exoscope, (c) transcranial and endoscopic skull base approaches, and (d) microvascular anastomosis on the human placenta and rats. The costs for human specimens and organization are supported by sponsors and participation fees. Each specimen preserved in a solution of alcohol, water, and softener can be reused for different courses, lowering the final cost. Considering the aforementioned checklist (Table 2), we have organized these courses taking into account the year of residency and related neurosurgical milestones. Each course included theoretical lectures and practical lab sessions.

### Basic techniques and approaches

The goal of these courses is to introduce the young resident to the basics of neurosurgery. Milestones of these courses include soft tissue dissection preserving the facial nerve and superficial temporal artery (STA), drilling techniques, basic craniotomies, sinus exposure, dura opening, interhemispheric and Sylvian fissure dissections, relevant intradural anatomy, and dura closure with a patch. During these courses, residents become familiar with instruments and techniques, can perform more common approaches such as pterional, parasagittal, and retrosigmoid approaches, and study surgical anatomy. Given the technological advancements in the neurosurgical field and the recent introduction of 3D exoscopes in surgical practice (112), we organize courses that not only use microscopes, but also hybrid microexoscopes and only exoscopes. The use of a 3D exoscope during a cadaveric course has gained widespread approval from trainees due to the limited presence of this technology in Italian hospitals.

### Transcranial and endoscopic skull base approaches

These courses are addressed to senior residents and neurosurgeons and consist of lectures and practice on skull base anatomy, pathologies, surgical strategies, and both transcranial and endoscopic approaches. Great emphasis is given to extradural anatomy and corridors. Participants exercise accurate drilling techniques and gentle intradural dissection in narrow and deep corridors. A combination of transcranial and endoscopic approaches can provide a 360° view of anatomy.

### Microvascular anastomosis on inert materials, placenta, and rats

The course is aimed at senior residents and neurosurgeons who want to learn the techniques of microsuturing and train their skills. Sutures, dedicated instruments, and step-by-step techniques are illustrated during lectures. In the lab, each participant has their own station with microscopes, instruments, and sutures (from 6.0 to 10.0). Starting from inert materials, like gauze and synthetic vessels, the participant can practice different anastomotic techniques on fresh human placenta, which simulates the vessels, arachnoid, and pia mater. The placenta is cannulated and perfused first with water and then with a colored saline solution (red for arteries and blue for veins). After practicing on *ex vivo* specimens and trying to perform a patent and functional anastomosis, the participant could perform it on *in vivo* rats under sedation. It is the most realistic model through which it is possible to simulate bloody dissection, blood pulsation, empathy, and anxiety with the aim of not killing the specimen.

Depending on specific areas of training, these presented milestones could be fine-tuned and expanded by training experts.

### Universities and international networks

Considering the aforementioned drawbacks, limitations, and differences among the neurosurgical training centers, in particular, in Europe and especially in our country (101, 109), that affect the learning curve of young neurosurgeons and ultimately the quality of medical assistance, the authors believe that it is time to renovate the residency systems. As previously proposed by Stienen et al., European standard guidelines for neurosurgical training could help further harmonize training among European countries and facilitate exchange (101). As regards the last decade, great work has been done by the European and Italian Neurosurgical Societies (EANS, SINCH) to contribute to the improvement of neurosurgical education through EANS training courses, the EANS spine diploma, SINCH basic courses for young residents, and hands-on courses. Nevertheless, all these opportunities remain at the discretion of the single resident and professor, are limited in time, and are affected by economical and organizational issues. The authors believe that the same compulsory theoretical program has to be suggested for all European countries during all the years of residency, in addition to being a parallel compulsory operative training plan that sustains and reinforces the surgical training in the OR with a uniform and verified number of procedures performed by the resident. Acquiring 3D anatomical knowledge, technical skills, manual dexterity, visuospatial ability, and surgical procedures should not be a choice for a neurosurgical resident, but rather a mandatory requirement to become a neurosurgeon. For this reason, the authors believe that each resident of all residency programs must have the opportunity to spend a period during each year of their residency in a cadaveric laboratory with a precise step-by-step “neurosurgical training checklist” with progressive milestones to improve the quality of operative training and guarantee equal skill acquisition all over the world.

However, high costs, difficult human specimen acquisition, and bureaucracy issues make this goal impossible. As reported by Italian anatomists (13), although Italian law is now more permissive about body donation, the number of centers performing anatomical dissections for the benefit of medical students and residents has decreased in favor of the few universities that can afford it.

## Limitations

Although human cadaveric specimens and fixed brain hemispheres are the most reliable models for reproducing anatomy and surgery on a living patient, technical limitations are not negligible. If embalmed specimens can be easily conserved, their soft tissue and brain stiffen, affecting the qualitative and quantitative study of the approach. On the contrary, fresh specimens overcome this issue, resulting in a greater similarity to the living one at the cost of limited conservation and use, increasing the costs. Second, the absence of the cerebrospinal fluid or the pathology of interest, such as tumors and vascular malformations, makes the cadaver defective and far from a real model.

Furthermore, the resident who spends some time in the laboratory should not forget that their experience in the OR is essential and irreplaceable for the emotional counterpart and the anxiety for responsibility related to the patient’s expectations.

## Future directions

As neurosurgeons are involved in intense cadaveric activities, we believe that more efforts should be made to create collaboration between universities and laboratories to optimize human, material and financial resources with the aim of establishing a solid and equitable operative training program. For this purpose, the provided “neurosurgery booklet” could be a starting point to be spread among universities and hands-on courses. The usefulness of this milestone approach should be discussed in the neurosurgical community and validated through questionnaires given to faculty experts and trainees. In this regard, we are formulating dedicated surveys for each level of competency to administer to participants of our cadaveric courses and research fellows in our lab. Similarly, training protocols on cadaveric specimens could be developed for spinal and peripheral neurosurgery. Although the higher costs of specimens and instrumentation could limit the possibilities, permanent laboratories for spinal and peripheral nerve surgery should be developed.

On another aspect, despite the great knowledge of neuroanatomy and surgical advances in the last few decades, descriptive neurosurgical anatomy is continually evolving through multidisciplinary description, new surgical corridors, and the improvement of minimally invasive skull base approaches (15). In this scenario, cadaver laboratories play a fundamental role in enhancing neuroanatomical knowledge and surgical outcomes. Different skull base research projects could be performed in the lab: description of anatomy with the integration of radiological examinations, development of three-dimensional models for surgical and training purposes, description of minimally invasive corridors, and comparison between surgical approaches. For this reason, a permanently equipped laboratory with research fellows is imperative.

## Conclusion

Microsurgical dissection of human cadaveric specimens is the optimal way to learn and train on neuroanatomy and neurosurgical procedures before safely performing them in the operating room. The authors believe that the neurosurgical preparation has to be integrated with a compulsory operative training program as a complementary activity during all the years of residency. The goals are acquiring three-dimensional anatomical knowledge, technical skills, manual dexterity, visuospatial ability, and surgical procedures. We provided a “neurosurgery training checklist” with progressive milestones for neurosurgical residents. This step-by-step operational program may improve the quality of training and guarantee equal skill acquisition across countries. We believe that more efforts should be made to create new microsurgical laboratories, popularize the importance of body donation, and encourage collaboration between universities and laboratories.

## Data Availability

The original contributions presented in the study are included in the article/Supplementary Material; further inquiries can be directed to the corresponding author.
